# Microtubule destabilization by SB225002 through preferential weakening of lateral tubulin interactions revealed by structural and computational analyses

**DOI:** 10.1016/j.jbc.2026.113625

**Published:** 2026-10-08

**Authors:** Xinyao Liu, Cuiyu Guo, Yuxi Wang, Dongsheng Lei

**Affiliations:** 1 School of Physical Science and Technology, Electron Microscopy Centre of Lanzhou University, Lanzhou University, Lanzhou, People's Republic of China; 2 Key Laboratory of Magnetism and Magnetic Functional Materials (Lanzhou University), Ministry of Education, Lanzhou, People's Republic of China; 3 Department of Pulmonary and Critical Care Medicine, Molecularly Targeted Research and Development Laboratory, Institute of Respiratory Health, West China Hospital, Sichuan University, Chengdu, Sichuan, China; 4 Frontiers Medical Center, Tianfu Jincheng Laboratory, Chengdu, Sichuan, China; 5 State Key Laboratory of Respiratory Health and Multimorbidity, West China Hospital, Sichuan University, Chengdu, Sichuan, China

**Keywords:** SB225002, tubulin, multisite binding, X-ray crystallography, molecular dynamics

## Abstract

Microtubules are essential for cell division and intracellular signaling, making them well-established targets for anticancer therapy. However, the clinical utility of conventional microtubule-targeting agents is often limited by toxicity and drug resistance. SB225002, originally identified as an interleukin-8 receptor antagonist, has been reported to disrupt microtubule dynamics and exhibit anticancer activity with relatively low toxicity in preclinical studies. Yet, the structural basis of its interaction with tubulin remains poorly understood. Here, we determined the crystal structure of the SB225002–tubulin complex at 2.60 Å resolution and performed molecular dynamics simulations to characterize ligand binding, as well as drug-induced changes in tubulin structure and inter-subunit interactions. The crystal structure revealed three SB225002 molecules occupying the colchicine- and vinblastine-binding regions of tubulin. Simulations showed persistent ligand association over the simulated timescale and relatively limited overall changes in longitudinal inter-subunit interactions, whereas structural flexibility and dynamic coupling were preferentially altered at the lateral-interface-associated surface of β-tubulin. Additional simulations incorporating both lateral and longitudinal tubulin interfaces further revealed a greater reduction in lateral than in longitudinal inter-subunit contacts upon drug binding. Together, these results support a mechanism in which SB225002 preferentially perturbs lateral tubulin interactions and provide a structural framework for understanding its microtubule-modulating activity.

Microtubules are essential components of the cytoskeleton that govern mitosis, intracellular transport, and signal integration 1, 2, 3, 4. Because of these roles, tubulin has long been established as a major target in anticancer drug discovery (5). Clinically, several microtubule-targeting agents (MTAs) including paclitaxel (6) and vinblastine (7) have been successfully used in the treatment of various malignancies. Colchicine and related compounds also represent a well-characterized class of tubulin-binding agents targeting the colchicine-binding site 8, 9. However, the clinical efficacy of many conventional MTAs is limited by systemic toxicity and the development of multidrug resistance (MDR), highlighting the need for new microtubule-targeting compounds with improved therapeutic profiles 10, 11, 12.

SB225002 is a small-molecule phenyl urea compound originally developed by White *et al.* in 1998 as a potent non-peptide antagonist of the interleukin-8 (IL-8/CXCL8) receptor, CXCR2 (Fig. 1*C*). (13). Initially, SB225002 was used to inhibit neutrophil chemotaxis, demonstrating notable anti-inflammatory and anti-migratory effects (14). Recently, accumulating evidence has revealed its antitumor activity. In various cancer models, SB225002 robustly suppressed tumor cell proliferation, migration, and invasion, while inducing cell cycle arrest and apoptosis (15). Importantly, further studies showed that SB225002 perturbs microtubule dynamics and induces mitotic catastrophe independently of its CXCR2 antagonism 16, 17. Molecular docking further suggested that SB225002 may bind to the vinca-binding region of β-tubulin (16). These findings indicate that the microtubule-targeting activity of SB225002 represents a mechanism distinct from its originally characterized CXCR2 antagonism and further support its potential as an anticancer compound.

**Figure 1:**
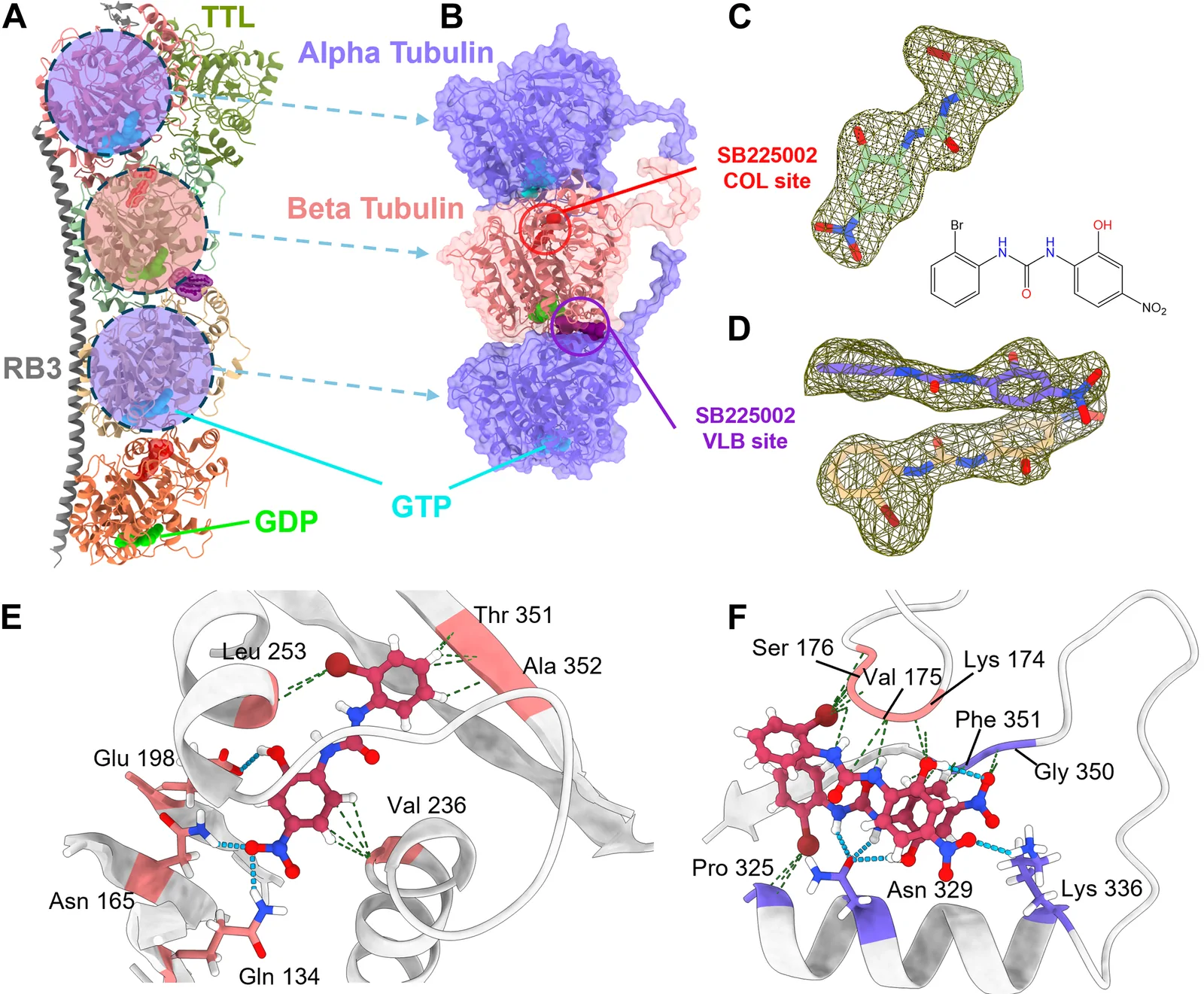
**Structural overview of the SB225002–tubulin complex and simulation model.***A*, crystal structure of the SB225002–tubulin complex showing the tubulin subunits and bound ligands. *B*, schematic representation of the three-subunit system used for molecular dynamics simulations, showing the spatial distribution of SB225002 molecules at the two binding sites. *C*, chemical structure and Polder mFo-DFc omit map of SB225002 at the colchicine (COL) site, shown in *yellow-green* and contoured at 5.0σ. *D*, Polder mFo-DFc omit map of SB225002 at the vinblastine (VLB) site, shown in *yellow-green* and contoured at 3.0σ. *E*, binding mode of SB225002 at the COL site, showing the surrounding protein region and key residues involved in hydrogen-bond formation. *F*, binding mode of SB225002 at the VLB site, showing neighboring structural elements and residues involved in hydrogen-bond interactions.

Compared with traditional natural product-derived MTAs, SB225002 has several potentially advantageous features. Its structurally simple scaffold is amenable to chemical modification, facilitating structure–activity relationship (SAR) studies and optimization. SB225002 has also shown relatively low toxicity and favorable selectivity in preclinical models, while retaining cytotoxic activity in cell lines exhibiting P-glycoprotein-mediated resistance and in cells with p53 deficiency or mutations 18, 19. Furthermore, its effects on both tubulin dynamics and the IL-8/CXCR2 signaling axis suggest that these distinct activities may jointly contribute to its anticancer effects.

Despite these promising features, high-resolution structural data elucidating SB225002’s interaction with tubulin are lacking. Previous computational studies of tubulin-targeting compounds have used molecular dynamics (MD) simulations to investigate ligand stability, key intermolecular interactions, and drug-induced conformational changes in tubulin 20, 21, 22, 23, 24. Molecular Mechanics/Poisson–Boltzmann Surface Area (MM/PBSA) calculations have also been applied to estimate the binding energetics of tubulin–ligand complexes 25, 26, 27. However, the structural and dynamic bases of SB225002 binding to tubulin remain poorly understood. Therefore, we aimed to determine the crystal structure of the SB225002–tubulin complex using X-ray crystallography. Based on this structure, we performed molecular dynamics simulations to characterize the binding mode, interaction interfaces, and conformational changes in tubulin. This combined structural and computational approach was designed to define the molecular basis of SB225002–tubulin recognition and its effects on tubulin structure and dynamics.

## Results

### SB225002 binding at the COL and VLB sites of β-tubulin in the experimental structure

The crystal structure of the protein–SB225002 complex was determined at a resolution of 2.60 Å using X-ray diffraction. Data collection and refinement statistics are summarized in Table 1. Structural analysis revealed three SB225002 molecules bound to β-tubulin (Fig. 1*A*). One SB225002 molecule occupied the colchicine (COL)-binding site and formed hydrogen bonds with Gln134, Asn165, and Glu198 of β-tubulin (Fig. 1*E*). The other two molecules were located within the vinblastine (VLB) binding site at the dimer interface, establishing hydrogen bonds with Lys174, Val175, and Ser176 of β-tubulin and Asn329, Lys336, and Phe351 of α-tubulin (Fig. 1*F*). Figure 1, *C* and *D* show the Polder omit maps of the SB225002 molecules at the two binding sites, respectively.

**Table 1:** Data Collection and Refinement Statistics for X-ray Structure

Data	Tubulin-SB225002
X-Ray Source	SSRF-BL18U1
Integration Package	HKL2000
Data Collection	
Resolution Range (Å)	45.29–2.60 (2.67–2.60)
Space Group	P 21 21 21
Unit Cell (Å, °)	104.68 157.35 180.64 (90 90 90)
Total Reflections	1,160,995
Unique Reflections	91,119
Redundancy	12.7 (5.5)
Completeness (%)	98.64 (76.4)
Mean I/sigma(I)	17.6 (1.7)
Rmerge	0.115 (0.909)
CC1/2	0.999 (0.619)
Structure Refinement	
R-factor/R-Free	0.2940/0.3341
Root Mean Square (RMS) (Bonds)	0.011
RMS (Angles)	1.231
No. of Atoms	17,712
Protein	17,234
Ligands	229
Waters	193
Average B-factor (Å^2^)	57.42
Protein	57.46
Waters	48.07
Ligands	61.75
SB225002-col	52.66
SB225002-vlb1	87.37
SB225002-vlb2	66.61
Others	56.14
Ramachandran Plot Statistics	
Most Favored Regions (%)	95.62
Allowed Regions (%)	4.29
Disallowed Regions (%)	0.09

To assess SB225002-induced conformational changes in β-tubulin, the drug-bound structure was structurally superimposed onto the apo structure (PDB ID: 4IIJ (28)). Residue-wise structural deviations following global superposition were then mapped onto the tubulin structure. Residue-wise structural deviations following global superposition were mapped onto the tubulin structure (Fig. 2*A*). Most regions showed only minor deviations, generally below 0.5 Å. In contrast, pronounced conformational differences were observed around the COL site, particularly in the T7 loop (Arg241–Asp249) (29), where the Cα positional deviations of residues Gly246–Asn249 exceeded 3.1 Å, with the largest deviation reaching approximately 6.4 Å at Asn249. Structural superposition further revealed a pronounced reorientation of the Gln245 and Asn247 side chains within the T7 loop, which adopted markedly different conformations in the drug-bound and apo structures (Fig. 2*D*). Notably, the position occupied by the Asn247 side chain in the apo structure overlaps with the bound SB225002 molecule in the complex structure, indicating that rearrangement of this side chain is required to accommodate the ligand.

**Figure 2:**
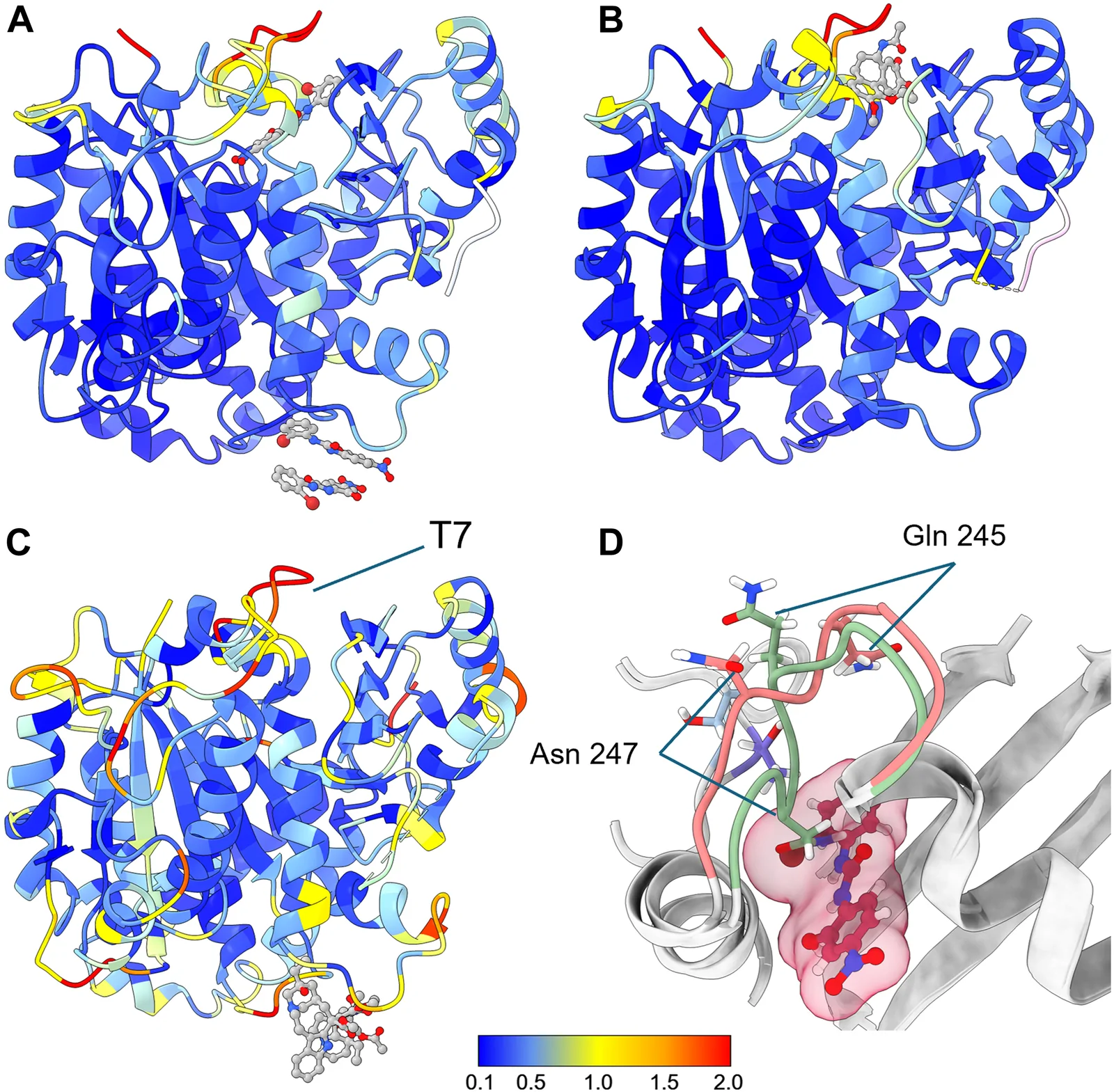
**Structural comparison of ligand-bound and apo β-tubulin.***A*, residue-wise structural deviations following superposition of the SB225002-bound structure onto the apo-tubulin structure (PDB ID: 4IIJ), mapped onto β-tubulin using a color scale. *B*, Corresponding structural-deviation map obtained by superimposing the colchicine-bound structure (PDB ID: 4O2B) onto the apo structure. *C*, corresponding structural-deviation map obtained by superimposing the vinblastine-bound structure (PDB ID: 1Z2B) onto the apo structure. *D*, close-up comparison of the T7 loop (Arg241–Asp249) in the SB225002-bound (*light red*) and apo (*sea green*) structures after global structural superposition.

The COL-bound (PDB ID: 4O2B (30)) and VLB-bound (PDB ID: 1Z2B (7)) tubulin structures were compared with the apo structure using the same structural-superposition and residue-wise deviation analysis described above. Colchicine binding was associated with pronounced conformational changes in the T7 loop (Fig. 2*B*), whereas vinblastine binding produced structural deviations in both the T7 loop and regions of the lateral surface of tubulin (Fig. 2*C*). Together, these comparisons indicate that rearrangement of the T7 loop is a common structural feature associated with ligand binding in this region, although the spatial extent of the conformational changes differs among compounds.

Although the experimentally determined structures provide direct information on drug–tubulin interactions, each crystal structure represents a conformational state stabilized under crystallization conditions and therefore cannot fully capture the dynamic behavior of tubulin. To complement the static structural analysis and characterize drug-induced changes in conformational dynamics, we performed molecular dynamics simulations of tubulin systems in the absence and presence of SB225002.

### Drug-induced perturbations at the β-tubulin lateral interface revealed by MD simulations

The temporal evolution of the Cα root-mean-square deviation (RMSD) for the first 425 residues of all tubulin subunits in the SB225002-bound and apo systems was monitored over three independent 250-ns MD simulations (Figs. 3*A* and S1). All β-tubulin subunits exhibited rapid convergence, with RMSD values stabilizing below 3.0 Å within the initial 25 ns, which was defined as the criterion for reaching stability. Specifically, the maximum RMSD observed for the β-tubulin subunits was approximately 2.5 Å, whereas the RMSD values for the α-tubulin subunits remained below 3.0 Å throughout the simulations. Time-dependent changes in the angle formed by the centers of mass of the three selected tubulin subunits were analyzed to monitor large-scale conformational shifts (Fig. 3, *C* and *F*). The average angle was consistent within the range of 165°–167° across all simulations, indicating that no significant longitudinal changes occurred in microtubule assembly during the simulations.

**Figure 3:**
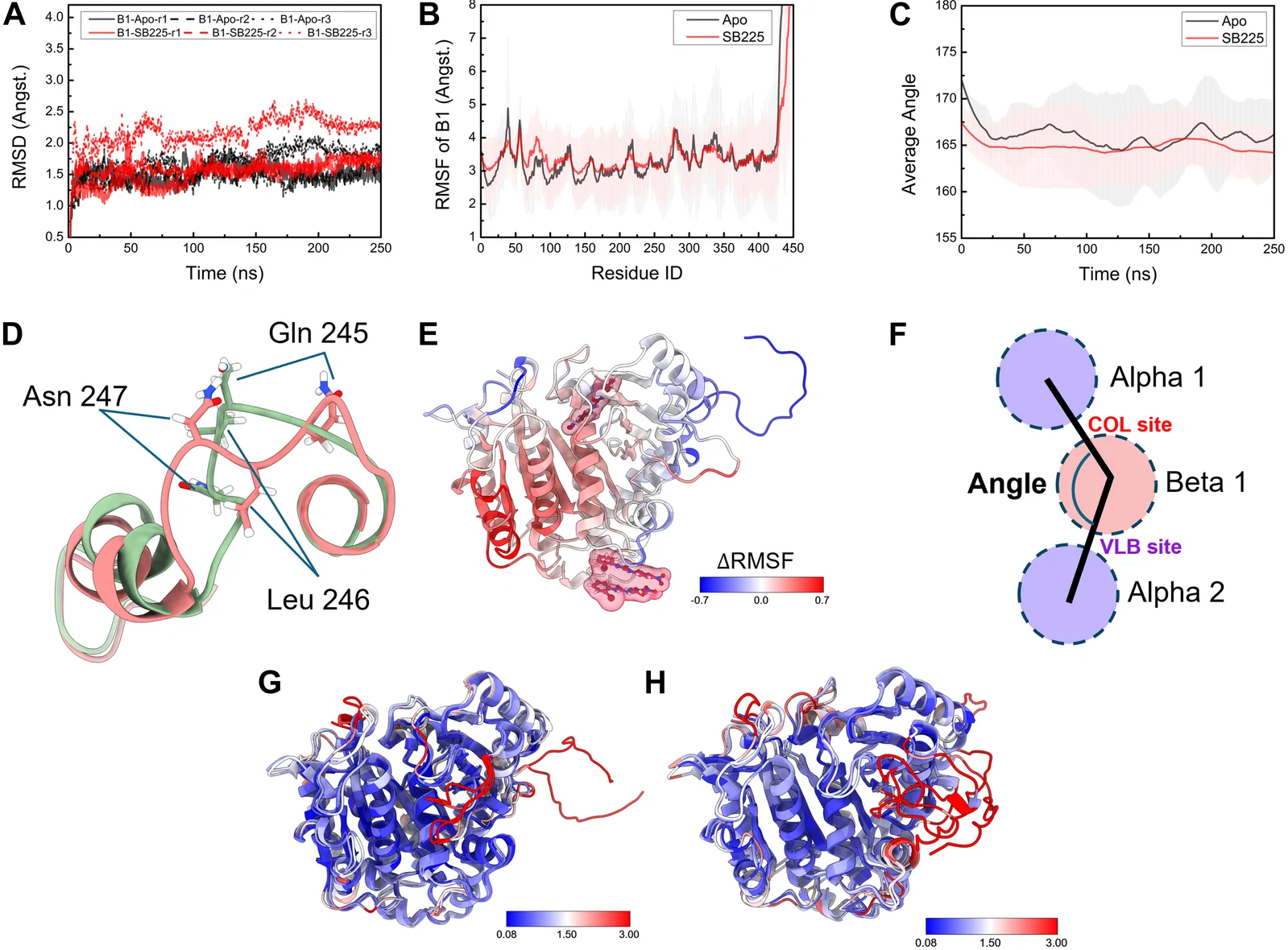
**Structural stability and conformational dynamics during molecular dynamics simulations.***A*, Cα root-mean-square deviation (RMSD) of residues 1 to 425 of β-tubulin during three independent 250-ns simulations of the apo and drug-bound systems. *B*, mean Cα root-mean-square fluctuation (RMSF) profiles of β-tubulin calculated from the three independent simulations under apo and drug-bound conditions. (*C*) Time evolution of the mean angle defined by the centers of mass of the three tubulin subunits, averaged across three independent simulations. *D*, close-up comparison of the T7 loop (Arg241–Asp249) in the average structures of the apo (*sea green*) and drug-bound (*light red*) systems. (*E*) Drug-induced changes in RMSF (ΔRMSF, drug-bound minus apo) mapped onto the β-tubulin structure. *F*, schematic definition of the angle analyzed in *panel* (*C*). *G and H*, average structures obtained from the equilibrated portions (50–250 ns) of three independent simulations of the apo (*G*) and drug-bound (*H*) systems. Cα positional deviations between each average structure and its corresponding initial structure after structural superposition are represented by color coding.

To further assess structural stability during the simulations, average structures from the 50 to 250 ns trajectory segment of each independent simulation were compared with their corresponding initial structures. Mapping of local Cα positional deviations showed that the structured core remained largely stable, whereas larger deviations were mainly observed in the reconstructed Leu273–Gln280 loop and the C-terminal regions (Fig. 3, *G* and *H*).

Consistent with the global RMSD analysis, most regions of both the apo and drug-bound systems showed only small deviations from their initial conformations, generally within approximately 1.5 Å. Larger deviations were mainly confined to the reconstructed loop region (Leu273–Gln280) and the C-terminal tails, which were absent or unresolved in the experimental structure and are intrinsically more flexible. These spatial distributions indicate that the conformational deviations observed during the simulations were localized primarily to flexible or reconstructed regions, whereas the structured core of tubulin remained largely stable.

Comparison of the average simulated structures further showed that the relative conformations of the T7 loop in the apo and drug-bound systems were consistent with the differences observed between the corresponding experimental structures (Fig. 3*D* *versus* Fig. 2*D*). This agreement indicates that the drug-associated T7-loop rearrangement observed crystallographically was retained during the simulations.

To further characterize local structural dynamics, root-mean-square fluctuation (RMSF) values were calculated separately for each simulation and then averaged across the three independent simulations for each system (Fig. 3*B*). Residues beyond position 425, corresponding to the highly flexible C-terminal tail, exhibited RMSF values consistently exceeding 10 Å and were excluded from subsequent comparative analyses, whereas RMSF values for the remaining residues were below 5 Å. Mapping the ΔRMSF values (drug-bound minus apo) onto the β-tubulin structure revealed that residues with pronounced increases in flexibility (ΔRMSF > 0.7 Å) were predominantly clustered at the lateral-interface-associated surface (Fig. 3*E*).

### Drug-binding positions and MM/PBSA binding energetics across three MD simulations

To evaluate the stability of SB225002 binding during the simulations, we examined the positional behavior of the bound ligands and used the MM/PBSA method to estimate their binding energetics at the COL and VLB sites.

Across the three independent simulations, SB225002 exhibited distinct dynamic behavior at the COL and VLB sites (Fig. 4*A*). The ligand at the COL site remained highly localized around its initial binding position throughout all three simulations (Fig. 4*B*), consistent with stable retention within the binding pocket. Its average MM/PBSA-estimated binding free energy was −46.37 kcal/mol (Fig. 4*C*).

**Figure 4:**
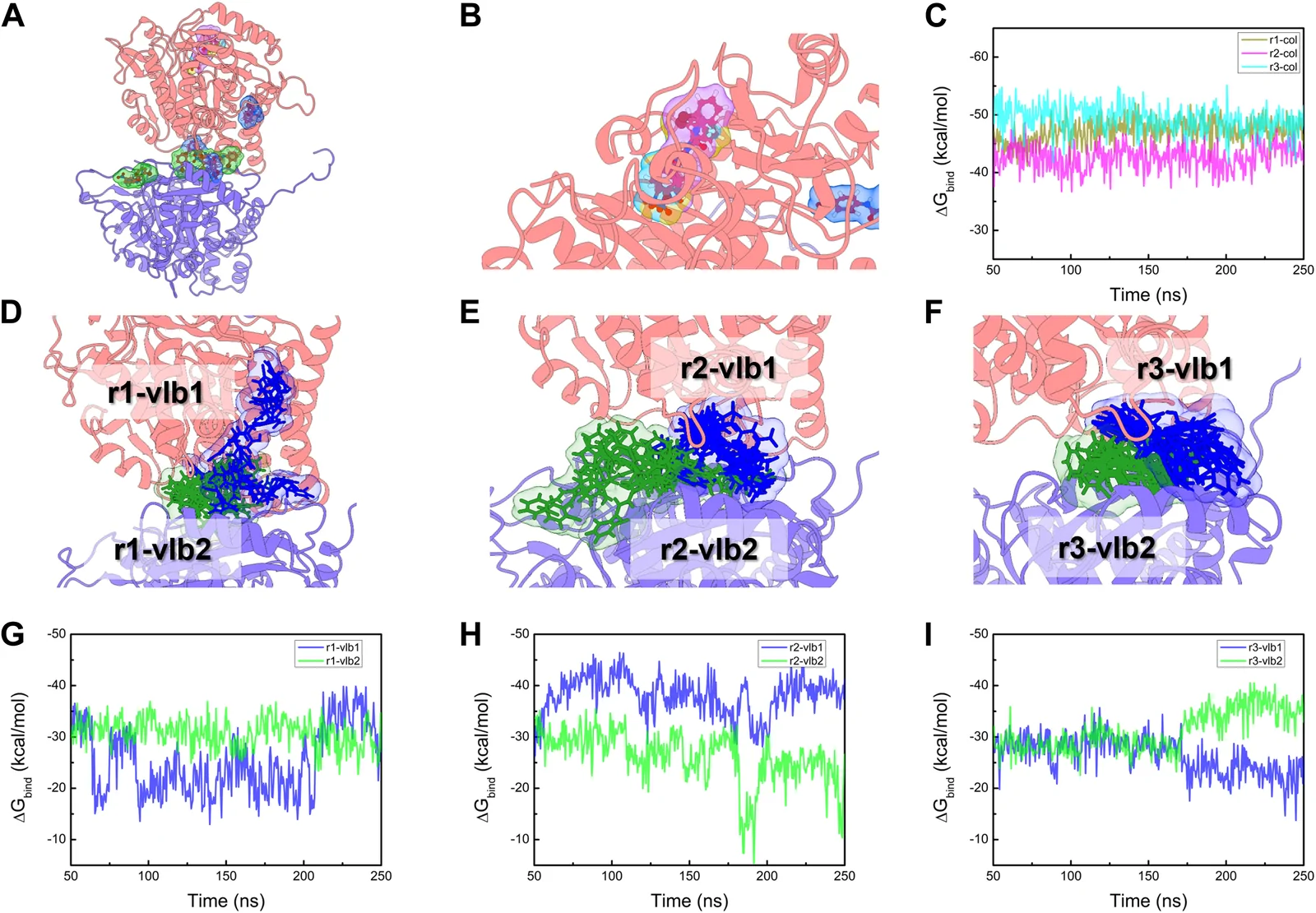
**Positional dynamics and MM/PBSA binding energetics of SB225002 during molecular dynamics simulations.***A*, representative binding positions of SB225002 observed across three independent simulations. Molecules with the same initial identity are indicated consistently. *B*, superimposed representative conformations of SB225002 at the COL site sampled during the three simulations. *C*, MM/PBSA-estimated binding free-energy profiles of SB225002 at the colchicine (COL) site. Curve colors correspond to the ligand representations in *panel* (*B*). *D–F*, superimposed representative conformations of the two SB225002 molecules at the VLB site during the first (*D*), second (*E*), and third (*F*) simulations. Thirteen conformations, including the initial structure, were sampled at approximately 20-ns intervals from each 250-ns trajectory. *G–I*, Corresponding MM/PBSA-estimated binding free-energy profiles of the two vinblastine (VLB) site ligands during the three simulations, with curve colors matching the ligand representations in *panels* (*D–F*).

In contrast, greater positional variability was observed for the two SB225002 molecules at the VLB site, designated vlb1 and vlb2. To visualize their positional ranges during the simulations, 13 representative conformations, including the initial structure, were sampled at approximately 20-ns intervals from each 250-ns trajectory and superimposed onto the corresponding tubulin structure (Fig. 4, *D*–*F*).

In the first simulation, vlb1 remained close to its initial position during the first half of the trajectory and subsequently shifted to a different position while remaining associated with β-tubulin, whereas vlb2 remained near its initial binding position (Fig. 4*D*). Consistent with this positional rearrangement, the MM/PBSA binding energy of vlb1 became less favorable during the transition and subsequently became more favorable, reaching −33.52 kcal/mol, whereas that of vlb2 remained comparatively stable, with an average value of −30.76 kcal/mol (Fig. 4*G*).

In the second simulation, vlb1 remained near its initial site, whereas vlb2 shifted toward α-tubulin while remaining in the vicinity of vlb1 (Fig. 4*E*). This positional rearrangement was accompanied by corresponding changes in its MM/PBSA energetic profile (Fig. 4*H*). In the third simulation, both VLB-site ligands remained close to their initial positions throughout the trajectory (Fig. 4*F*). Although their individual MM/PBSA energies fluctuated over time, their combined energetic contribution remained comparatively stable (Fig. 4*I*).

For simulations in which both VLB-site ligands remained associated with the binding region, their combined average MM/PBSA binding energy was −59.14 kcal/mol. Overall, the VLB-site ligands displayed greater conformational mobility than the COL-site ligand, but all SB225002 molecules remained associated with tubulin over the simulated timescale, with no irreversible ligand dissociation observed.

### Drug effects on longitudinal interactions between tubulin dimers

To evaluate the effects of SB225002 on inter-residue interactions within the simulated tubulin assembly, salt-bridge occurrence and hydrogen-bonding interactions were compared between the apo and the drug-bound systems.

#### Salt bridge analysis between adjacent tubulin subunits

Among the salt bridges examined, Lys103–Glu401, Lys174–Glu205, and Asp295–Lys297 showed the largest decreases in occurrence upon drug binding, from 42.28% to 34.57%, 46.90% to 22.35%, and 67.33% to 47.44%, respectively (Fig. 5*A*). Of these, the Lys174–Glu205 salt bridge is located at the longitudinal interface between adjacent tubulin subunits and exhibited the largest relative decrease.

**Figure 5:**
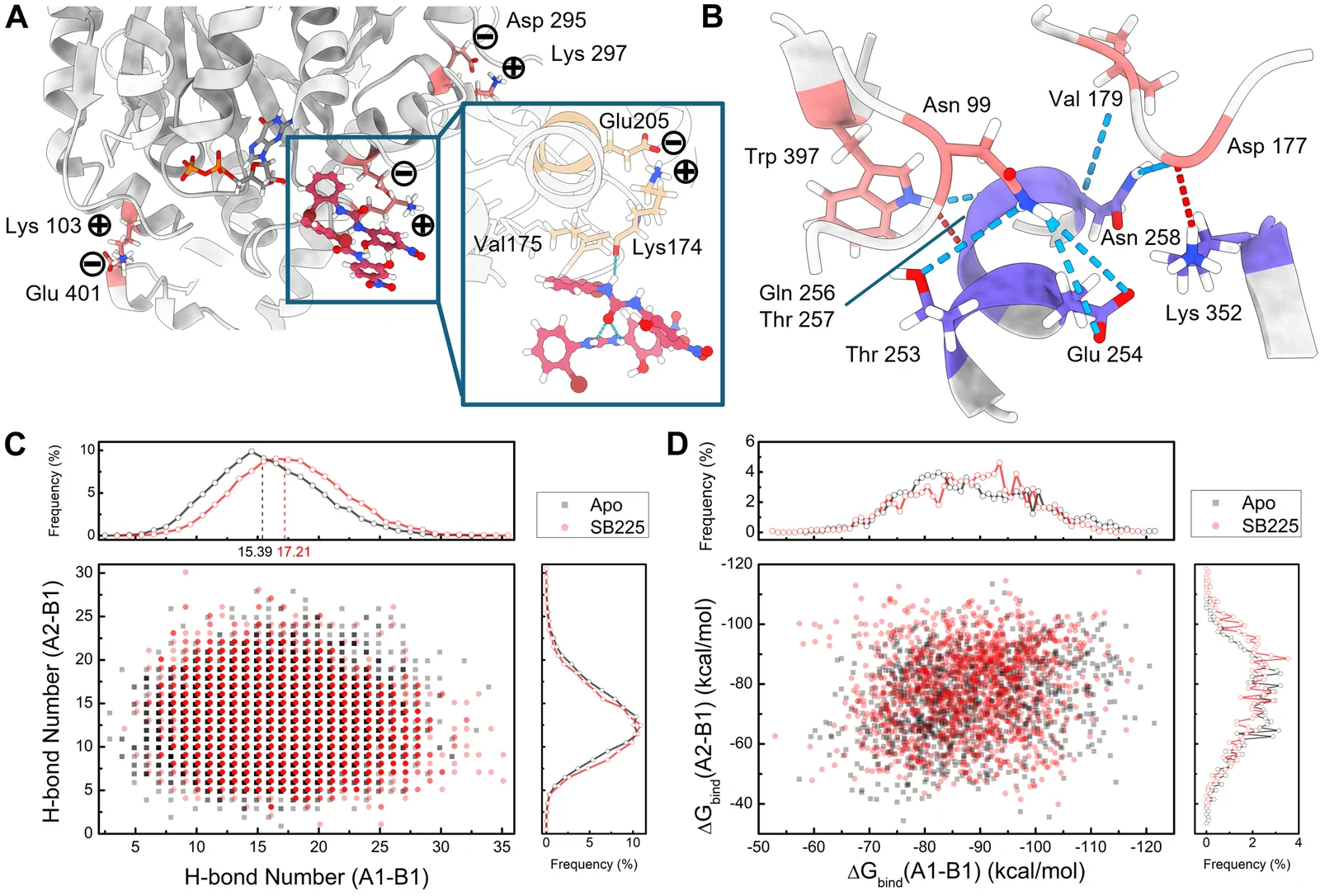
**Effects of SB225002 on longitudinal tubulin interactions.***A*, structural locations of the three salt bridges showing the largest changes in occurrence upon SB225002 binding. The inset highlights local interactions involving SB225002 near the Lys174–Glu205 salt bridge. *B*, hydrogen-bonding interactions at the longitudinal tubulin interface. *Red* and *blue dashed lines* indicate hydrogen bonds with increased and decreased occurrence, respectively, upon drug binding. *C*, joint distributions of the numbers of hydrogen bonds at the A1–B1 intradimer and A2–B1 interdimer interfaces under apo and drug-bound conditions. Each point represents paired values from the same simulation frame, with data from three independent simulations pooled for visualization; marginal curves show the corresponding frequency distributions. *D*, joint distributions of the MM/PBSA-estimated binding free energies at the A1–B1 and A2–B1 interfaces, displayed in the same manner as in panel (*C*).

Structural analysis suggested that the reduction in Lys174–Glu205 salt-bridge formation was associated with local interactions involving SB225002. The ligand formed persistent hydrogen-bonding interactions with Lys174 and Val175 (inset, Fig. 5*A*), which altered the local interaction environment around Lys174 and may have reduced its availability for salt-bridge formation with Glu205. In addition, SB225002 occupied a position spanning the β-tubulin and adjacent α-tubulin regions (Fig. 1*F*), allowing simultaneous interactions with residues from both subunits. These local rearrangements were accompanied by a reduction of over 50% in the occurrence of the Lys174–Glu205 salt bridge.

The other two salt bridges showing reduced occurrence, Lys103–Glu401 and Asp295–Lys297, are located at the lateral-interface-associated surface of β-tubulin. Their altered frequencies therefore indicate that SB225002 also affects local interactions in regions associated with lateral tubulin contacts, which were examined further in subsequent analyses.

#### Hydrogen bond analysis at the longitudinal interface

The hydrogen-bonding network at the longitudinal tubulin interfaces was further analyzed, with particular attention to the region surrounding the VLB-binding site. In Figure 5*B*, blue and red dashed lines indicate hydrogen bonds whose occurrence decreased and increased, respectively, upon drug binding.

To quantify longitudinal interactions, the numbers of hydrogen bonds at the A1–B1 intradimer interface and the A2–B1 interdimer interface were analyzed jointly (Fig. 5*C*). Each point represents paired values from the same simulation frame, with data from the three independent simulations pooled for visualization; the marginal curves show the corresponding frequency distributions. SB225002 binding was associated with an average increase of 1.8 hydrogen bonds at the A1–B1 intradimer interface, whereas the A2–B1 interdimer hydrogen-bond distribution remained largely unchanged.

The MM/PBSA-estimated binding free energies of the A1–B1 and A2–B1 interfaces were analyzed in the same manner (Fig. 5*D*). No substantial drug-induced change was observed at the A2–B1 interdimer interface. Together, these results indicate that SB225002 induces local rearrangements within the longitudinal assembly without substantially altering the overall interaction between adjacent tubulin dimers.

### Drug-induced changes in lateral interactions

As the crystal structures and microtubule models used in this study contained only four longitudinally arranged tubulin subunits, direct analysis of drug-induced changes in lateral interactions was impossible. Instead, we indirectly assessed the potential effects by comparing β-tubulin dynamics from our simulations with cryo-EM structures through structural alignment and positional deviation analyses. Although this approach cannot fully replicate native microtubule lattices, it offers valuable insights into drug-induced changes in lateral contacts.

The cryo-EM structure of apo-microtubules (PDB: 8OTZ (31)) was selected for the comparison. A cross-sectional view of this structure is shown in Figure S2*A*, with the interface indices between adjacent β-tubulin subunits labeled 1 to 15. The corresponding hydrogen-bond interactions at these interfaces are presented in Figure S2*C*. Hydrogen bonds between adjacent β-tubulin subunits were primarily localized to three residue regions: 85 to 125, 278 to 297 and 336. (Fig. S2*B*).

### DCCM reveals altered dynamics associated with the lateral interface

To characterize the effects of SB225002 on the internal dynamics of β-tubulin, we calculated dynamic cross-correlation matrices (DCCMs) for the apo and drug-bound systems. The DCCMs from three independent simulations per condition are shown in Figure S3, *A* and *B*, and the corresponding averaged DCCMs are shown in Figure 6, *A* and *B*. Pronounced changes upon drug binding were observed in the regions outlined by black boxes.

**Figure 6:**
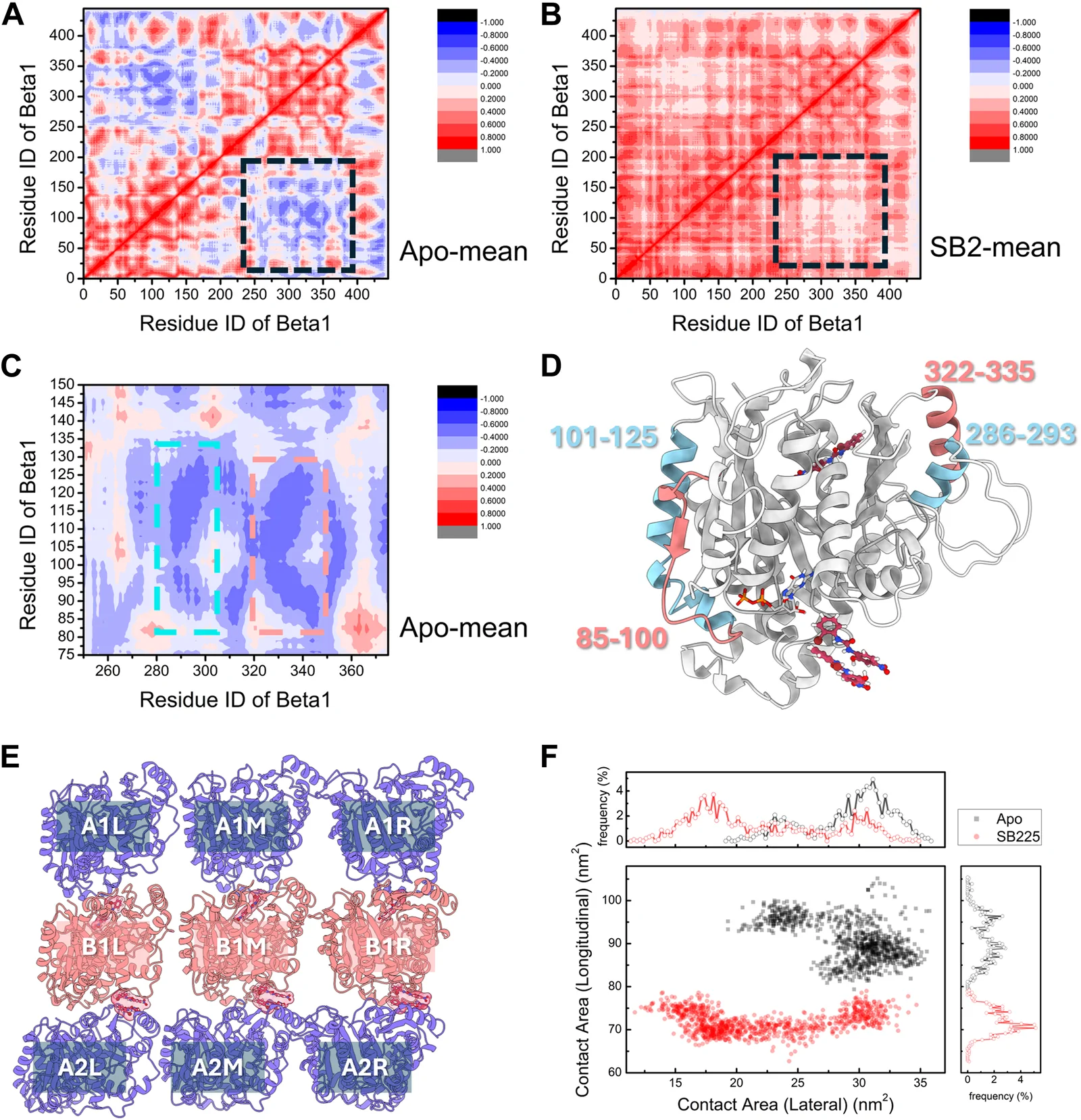
**Dynamic perturbations associated with the lateral interface and comparison of lateral and longitudinal contacts.***A and B*, average dynamic cross-correlation matrices (DCCMs) of β-tubulin under apo (*A*) and drug-bound (*B*) conditions. Black boxes indicate regions showing pronounced drug-induced changes. *C*, enlarged view of the boxed region in the apo DCCM, highlighting residue groups exhibiting pronounced anti-correlated motions. *D*, structural mapping of the residue groups identified in panel (*C*) onto β-tubulin. *E*, the 3 × 3 tubulin model used to evaluate lateral and longitudinal inter-subunit contacts, with individual rows and columns labeled accordingly. *F*, temporal profiles and frequency distributions of the summed lateral and longitudinal contact areas in the drug-free and drug-bound 3 × 3 models.

In the apo system, these regions exhibited strong negative correlations, indicating pronounced anti-correlated motions between the corresponding structural elements (Fig. 6, *A* and *C*). Upon drug binding, the correlations shifted toward zero or weakly positive values (Fig. 6*B*), indicating a marked reduction in the pre-existing anti-correlated dynamic coupling. Detailed analysis identified two residue-group pairs, residues 85–100 and 322–335, and residues 101–125 and 286–293, that showed particularly strong anti-correlated motions in the apo state (Fig. 6*C*). Structural mapping revealed that these residues are located at the lateral-interface-associated surface of β-tubulin and overlap with regions involved in hydrogen-bond formation at the lateral interface (Figs. 6*D* and S2, *B*). These results indicate that SB225002 preferentially perturbs the intramolecular dynamics associated with the lateral interface. However, because the crystal-structure-based model contains only three longitudinally arranged tubulin subunits, it does not include lateral inter-subunit contacts and therefore cannot directly assess whether these dynamic perturbations translate into altered lateral interactions.

### Comparison of lateral and longitudinal interactions in the 3 × 3 tubulin model

To directly evaluate the effects of SB225002 on lateral tubulin–tubulin contacts, we constructed larger 3 × 3 tubulin models representing the drug-free and drug-bound states. The cryo-EM structure (PDB ID: 8OTZ) was used as the reference framework, with structural information from the crystal structure (PDB ID: 24PT) incorporated into the drug-bound model (Fig. 6*E*). The detailed modeling procedure is described in the Methods section. Three independent 150-ns MD simulations were performed for each system, and all subunits reached stable conformational states after approximately 20 ns (Fig. S4).

Contact area was used to compare lateral and longitudinal inter-subunit interactions because interaction energies cannot be readily decomposed into independent lateral and longitudinal components, particularly in the presence of interactions between diagonally positioned subunits. Contact areas were therefore calculated between adjacent rows and columns. To reduce interface-specific variations and improve the robustness of the comparison, the values were summed over the three subunits within each row or column.

Specifically, the lateral contact area was defined as the total contact area between the middle (M) column and the adjacent left (L) and right (R) columns, whereas the longitudinal contact area was defined as the total contact area between the row containing the three β-tubulin subunits (B1) and the adjacent rows containing A1 and A2. The temporal profiles and frequency distributions are shown in Figure 6*F*, and the corresponding uncombined contact-area data are provided in Figure S3, *C* and *D*.

Drug binding shifted both longitudinal and lateral contact-area distributions toward smaller values (Fig. 6*F*). The mode of the longitudinal distribution decreased from 88.87 to 70.37 nm^2^, corresponding to a reduction of approximately 20%, whereas the mode of the lateral distribution decreased from 31.12 to 18.125 nm^2^, a reduction of over 40%. Thus, the relative reduction in the modal lateral contact area was approximately twice that observed in the longitudinal direction.

The time-averaged contact areas showed the same trend: the longitudinal and lateral contact areas decreased by 20.75% and 24.84%, respectively, upon drug binding. Although the difference was less pronounced for the time-averaged values, lateral contacts remained more strongly affected. Together with the lateral-interface-associated dynamic perturbations identified in the crystal-structure-based model, these results support a preferential effect of SB225002 on lateral tubulin interactions.

## Discussion

The combined structural and simulation analyses support a mechanism in which SB225002 preferentially perturbs lateral rather than longitudinal tubulin interactions. The crystal-structure-based model provided a direct experimental basis for identifying local drug-induced changes and showed relatively limited overall effects on longitudinal interactions, whereas structural flexibility and intramolecular dynamic coupling were preferentially altered at the lateral-interface-associated surface of β-tubulin. The larger 3 × 3 model further allowed lateral and longitudinal contacts to be evaluated directly, independently supporting the same trend.

The 3 × 3 model nevertheless remains an approximation of the native microtubule lattice and incorporates structural information from different experimental structures. Its quantitative results should therefore be interpreted primarily as semi-quantitative support for the relative effects of SB225002 rather than as precise estimates of physiological changes. Despite these limitations, the agreement between the two modeling approaches strengthens the conclusion that preferential weakening of lateral tubulin interactions is an important component of the mechanism of action of SB225002.

## Experimental procedures

### Special reagents

SB225002 (Alexis Biochemicals, Enzo Life Sciences Inc.) was dissolved in anhydrous dimethyl sulfoxide (DMSO; Sigma-Aldrich), aliquoted, and stored at −80 °C, according to the manufacturer’s instructions. Porcine brain tubulin was isolated from the cytoskeleton.

### Protein expression and purification

The T2R-TTL complex, composed of *α*-tubulin, *β*-tubulin, one stathmin-like domain of RB3 (RB3-SLD), and one tubulin tyrosine ligase (TTL), was synthesized using methods previously described in the literature 32, 33. Porcine brain tubulin (from Cytoskeleton, Catalog # T-238P) was diluted to a concentration of 10 mg/ml in a buffer solution (80 mM Pipes, 2.0 mM MgCl_2_, 0.5 mM EDTA, and 1 mM guanosine-5′-triphosphate [GTP], pH 6.9) and then stored at −80 °C for later use. The RB3–SLD protein was overexpressed in *Escherichia coli* strain BL21 (DE3) and subjected to primary purification *via* anion-exchange chromatography (QFF; GE Healthcare), followed by gel filtration (Superdex 75; GE Healthcare). The purified RB3–SLD protein was concentrated to 10 mg/ml and stored at −80 °C. TTL was overexpressed in *E. coli* strain BL21 (DE3) and purified using nickel-affinity chromatography and subsequent gel filtration (Superdex 200; GE Healthcare). The purified TTL was concentrated in a solution containing Bis-Tris propane, 200 mM NaCl, 2.5 mM MgCl_2_, 5 mM β-mercaptoethanol, and 1% glycerol (pH 6.5) to 20 mg/ml and stored at −80 °C. The purity of both RB3 and TTL was verified using sodium dodecyl sulfate-polyacrylamide gel electrophoresis and Coomassie Brilliant Blue staining. To assemble the T2R-TTL complex, tubulin, RB3-SLD, and TTL were combined in a solution (5 mM tyrosine, 10 mM DTT, and 1 mM β,γ-methyleneadenosine 5′-triphosphate disodium salt) maintaining a mass ratio of 1:1.2:1.3, and the resulting complex was concentrated to 20 mg/ml at 4 °C.

### Crystallization, data collection, and processing

Crystals of the T2R-TTL complex were successfully grown using the hanging drop vapor diffusion method at 20 °C. The complex was mixed with a solution containing 6% PEG4000 (polyethylene glycol 4000), 8% glycerol, 0.1 M MES (pH 6.7), 30 mM CaCl_2_, and 30 mM MgCl_2_. The seeding method facilitated the formation of single crystals. Crystals began to appear after 3 days of incubation and reached their maximum dimensions within 5 days. For the crystal soaking procedure, 0.1 μl of SB225002 (10 mM in DMSO) was added to a 2 μl crystal solution and incubated for 5 h at 20 °C prior to data collection.

The crystals were briefly soaked in a cryoprotectant solution containing 20% (v/v) glycerol, mounted on nylon loops, and flash-cooled in liquid nitrogen. Diffraction data were collected using the beamline BL18U1 at the Shanghai Synchrotron Radiation Facility, Shanghai, China. Data were processed, indexed, integrated, and scaled using the HKL2000 software suite (34).

The structure of the T2R–TTL–SB225002 complex was determined by molecular replacement using PHASER, with the T2R–TTL structure (PDB ID: 4I55 (32)) as the search model. The initial model and geometric restraint parameters for SB225002 were generated using PRODRG (35). Manual model building was performed using COOT (36), and crystallographic refinement was carried out using phenix.refine (37). Model quality was assessed using MolProbity (38). Molecular structure figures were prepared using UCSF ChimeraX (39).

### Structural comparison of experimental structures

The drug-bound and apo β-tubulin structures were structurally superimposed using the Matchmaker tool in UCSF ChimeraX, using all Cα atoms of β-tubulin, with the apo structure as the reference.

### Microtubule structure preparation for MD simulations

In this study, two structural models based on X-ray crystal structures were developed for three-subunit MD simulations. The drug-bound model was constructed from the crystal structure determined in this study, whereas the apo model was derived from the apo-tubulin crystal structure (PDB: 4IIJ (28)). To model the missing β-tubulin loop region (Leu273–Gln280) in the drug-bound structure, MODELLER was used to generate five alternative conformations, from which the lowest-energy structure without steric clashes was selected (40). The C-terminal tails were absent in both crystal structures (Val437–Glu450 of α-tubulin and Thr429–Ala445 of β-tubulin), consistent with the models generated by MODELLER. In both systems, three longitudinally arranged tubulin subunits were selected, consisting of one β-tubulin sandwiched between two α-tubulin subunits (Figs. 1, *B* and *F*).

To enable analysis of lateral tubulin–tubulin interactions, two additional nine-subunit models were constructed in a 3 × 3 arrangement, representing the apo and drug-bound states (Fig. 6*E*). The apo model was derived from the apo microtubule cryo-EM structure (PDB: 8OTZ). Nine tubulin subunits were selected and designated A1L, A1M, and A1R in the upper row; B1L, B1M, and B1R in the middle row; and A2L, A2M, and A2R in the lower row, where L, M, and R denote the left, middle, and right lateral positions, respectively. A1 and A2 correspond to α-tubulin subunits, whereas B1 corresponds to β-tubulin. Incomplete α-tubulin subunits in the selected cryo-EM model were replaced with the complete A1M α-tubulin structure from the same model after structural superposition.

Because a corresponding drug-bound nine-subunit cryo-EM structure was unavailable, the drug-bound model was generated by structural superposition. A longitudinal three-subunit unit containing the bound ligand was taken from the drug-bound structure (PDB: 24PT) and independently aligned, using the β-tubulin subunit as the reference, to the B1L, B1M, and B1R positions of the apo nine-subunit model. The resulting lateral interfaces were inspected to confirm the absence of severe steric clashes or atomic overlaps.

Hydrogen atoms were added to all systems using the PSFgen plugin in VMD (41). The three-subunit systems contained two α-tubulins and one β-tubulin, with two GTP molecules bound to the α-tubulins and one guanosine-5′-diphosphate (GDP) molecule bound to the β-tubulin (Fig. S5). The apo three-subunit model was solvated in a water box of 105.5 × 139.9 × 180.3 Å^3^, whereas the drug-bound model was solvated in a water box of 106.9 × 125.8 × 183.1 Å^3^, with a minimum distance of 20 Å between the protein surface and the box boundaries. A total of 84 Na^+^ ions were added to neutralize each system, followed by additional Na^+^ and Cl^−^ ions to achieve a final salt concentration of 0.15 mol/L.

The nine-subunit systems were solvated and ionized using the same procedure, with a minimum protein-to-box distance of 10 Å. The apo nine-subunit model was solvated in a water box of 112.7 × 166.6 × 187.9 Å^3^ and neutralized with 187 Na^+^ ions, whereas the drug-bound model was solvated in a water box of 104.1 × 168.0 × 185.2 Å^3^ and neutralized with 179 Na ^+^ ions. Additional Na^+^ and Cl^−^ ions were then added to both systems to reach a final salt concentration of 0.15 mol/L. All system components were assembled using VMD.

To minimize system energy, positional restraints of 10 kcal mol^−1^ Å^−2^ were applied to the protein backbone atoms and ligand non-hydrogen atoms. All simulations were performed using NAMD 2.13 with a 2 fs timestep and a 12 Å cutoff for nonbonded interactions (42). Following 20,000 steps of energy minimization, the system was gradually heated from 20 to 310 K by increasing the temperature by 10 K every 500 steps. Temperature was controlled using Langevin dynamics with a damping coefficient of 5 ps^−1^. The system was subsequently equilibrated for 0.75 ns at 310 K and 1 atm, with pressure maintained using the Langevin piston method. Positional restraints were then progressively reduced over 0.8 ns, after which the three-subunit systems were subjected to 250 ns of unrestrained production MD simulations, whereas the nine-subunit systems were simulated for 150 ns under the same conditions.

Force-field parameters for the proteins, ions, GDP, and GTP were assigned using the CHARMM36 force field (43), and water molecules were represented using the TIP3P water model (44).

Because force-field parameters for SB225002 were not available in the existing parameter database, an initial parameter set was generated based on the CGenFF assignment provided by CHARMM-GUI and subsequently refined using the Force Field Toolkit (FFTK) plugin in VMD 45, 46. Molecular geometry optimization and bonded-parameter refinement were performed at the MP2/6-31G∗ level, with dihedral parameters fitted against quantum-mechanical potential energy scans at the same level of theory. Partial atomic charges were also evaluated using HF/6-31G∗ calculations during the FFTK parameterization procedure; however, the CGenFF-assigned charges were retained in the final model because preliminary aqueous simulations using the FFTK-derived charges showed substantial deviations from the expected ligand conformation. Van der Waals parameters were adopted from the corresponding CGenFF atom types.

### Average-structure and positional-deviation analyses

For each of the three independent simulations of the apo and drug-bound systems, average structures were generated from the 50 to 250 ns trajectory segment, which was selected to conservatively exclude the initial stabilization period. After structural fitting of the selected trajectory frames, average Cα coordinates were calculated using the “measure avpos” command in VMD. Each resulting average β-tubulin structure was then structurally superimposed onto the corresponding initial β-tubulin structure using the Matchmaker tool in UCSF ChimeraX, using all Cα atoms of β-tubulin. Local Cα positional deviations between the superimposed average and initial structures were then calculated and mapped onto the structure.

### Binding-free energy calculation

The binding-free energies between the ligand and tubulin, as well as between adjacent tubulin subunits, were estimated using the MM/PBSA method 47, 48 implemented in the CaFE package within VMD (49). For the three-subunit systems, approximately 400 frames were extracted at 500 ps intervals from the final 200 ns of the 250 ns unrestrained production trajectories. Unless otherwise specified, MM/PBSA calculations were performed using the default parameters implemented in CaFE, with APBS employed for the Poisson–Boltzmann calculations (50). Detailed calculation settings, including the internal dielectric constant, are provided in the Supporting Information. The binding-free energy was calculated as the sum of the molecular mechanics energy and the solvation free energy. The entropic contribution (−TΔS) was not included in the present analysis.

### Calculation of DCCM

To analyze residue-residue motion patterns and intramolecular interactions within tubulin, a dynamic cross-correlation matrix was computed using the dccm() function in the Bio3D package 51, 52. DCCM quantitatively describes the correlated and anticorrelated motions between residue pairs over the simulation trajectory, revealing possible allosteric pathways and cooperative motions within the tubulin structure (53).

### Contact area calculation

The contact area between adjacent tubulin subunits was estimated from the solvent-accessible surface area (SASA) according to

$$Contact\;Area=\frac{{SASA}_{1}+{SASA}_{2}-{SASA}_{12}}{2}$$

where (${SASA}_{1}$) and (${SASA}_{2}$) are the solvent-accessible surface areas of the individual subunits and ${SASA}_{12}$ is that of the corresponding subunit pair. A solvent probe radius of 1.4 Å was used for the SASA calculations.

## Data availability

The datasets used and analyzed in this study are available from the corresponding author upon request. The atomic coordinates and structure factors for the SB225002-tubulin complex have been deposited in the Protein Data Bank (PDB) under accession code 24PT.

## Supporting information

This article contains supporting information (50).

## Conflict of interest

The authors declare that they have no conflicts of interest with the contents of this article.
