# Supplementary material for: Microtubule destabilization by SB225002 through preferential weakening of lateral tubulin interactions revealed by structural and computational analyses
Source: J Biol Chem. 2026 Oct 8;302(11):113625. doi: 10.1016/j.jbc.2026.113625 (PMC13755465; doi:10.1016/j.jbc.2026.113625)
Supplement: Supplementary file 1 [file mmc1.pdf]

Supporting Information

Microtubule destabilization by SB225002 through preferential weakening of lateral tubulin interactions revealed by structural and computational analyses

Xinyao Liu[1,2,‡,*], Cuiyu Guo[3,4,5,‡], Yuxi Wang[3,4,5,*], and Dongsheng Lei[1,2,*]

[1]School of Physical Science and Technology, Electron Microscopy Centre of Lanzhou University, Lanzhou University, Lanzhou 730000, People's Republic of China

[2]Key Laboratory of Magnetism and Magnetic Functional Materials (Lanzhou University), Ministry of Education, Lanzhou, People's Republic of China

[3]Department of Pulmonary and Critical Care Medicine, Molecularly Targeted Research and Development Laboratory, Institute of Respiratory Health, West China Hospital, Sichuan University, Chengdu, 610041, Sichuan, China

[4]Frontiers Medical Center, Tianfu Jincheng Laboratory, Chengdu, 610212, Sichuan, China

[5]State Key Laboratory of Respiratory Health and Multimorbidity, West China Hospital, Sichuan University, Chengdu, 610041, Sichuan, China

[‡]Denotes equal authorship contribution.

*Authors to whom correspondence should be addressed: liuxy2020@lzu.edu.cn, yuxiwang@scu.edu.cn, leids@lzu.edu.cn.

Content

Figure S1. Cα root-mean-square deviation (RMSD) profiles of individual tubulin subunits in the three-subunit model. S-3

Figure S2. Lateral hydrogen-bonding interfaces in β-tubulin. S-4

Figure S3. Independent dynamic cross-correlation matrices (DCCM) analyses and uncombined contact-area distributions. S-5

Figure S4. Cα root-mean-square deviation (RMSD) profiles of individual tubulin subunits in the 3 × 3 models. S-6

Figure S5. Chemical structures of tubulin-bound nucleotides. S-6

MM/PBSA Calculation Parameters and Details S-7

Figure S6. Effect of the internal dielectric constant on MM/PBSA-estimated binding free energies. S-7

Table S1. Key parameters used in the MM/PBSA calculations. S-8

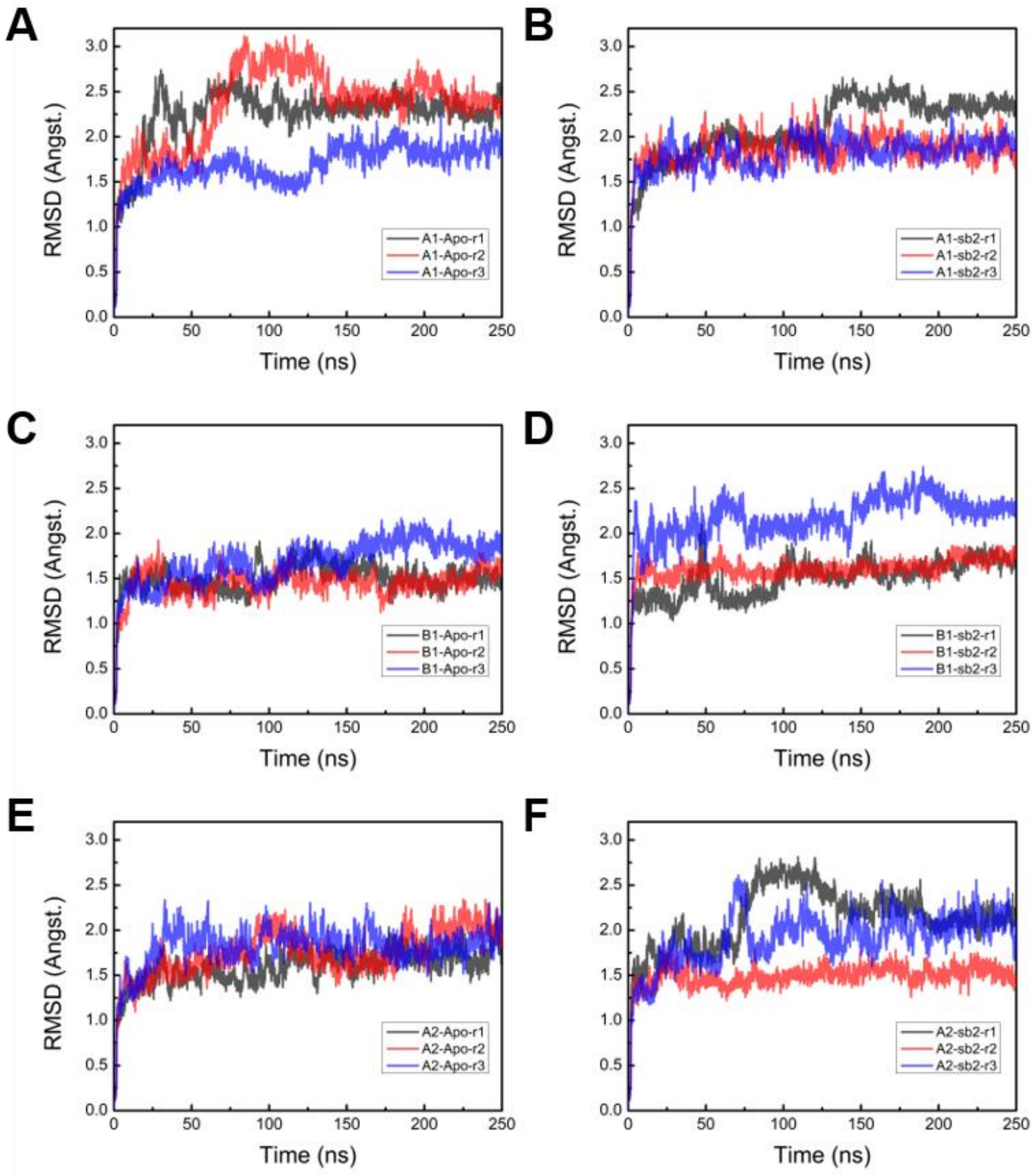

Figure S1. Cα root-mean-square deviation (RMSD) profiles of individual tubulin subunits in the three-subunit model. Cα RMSD values for residues 1–425 of each tubulin subunit during three independent 250-ns simulations under apo and drug-bound conditions.

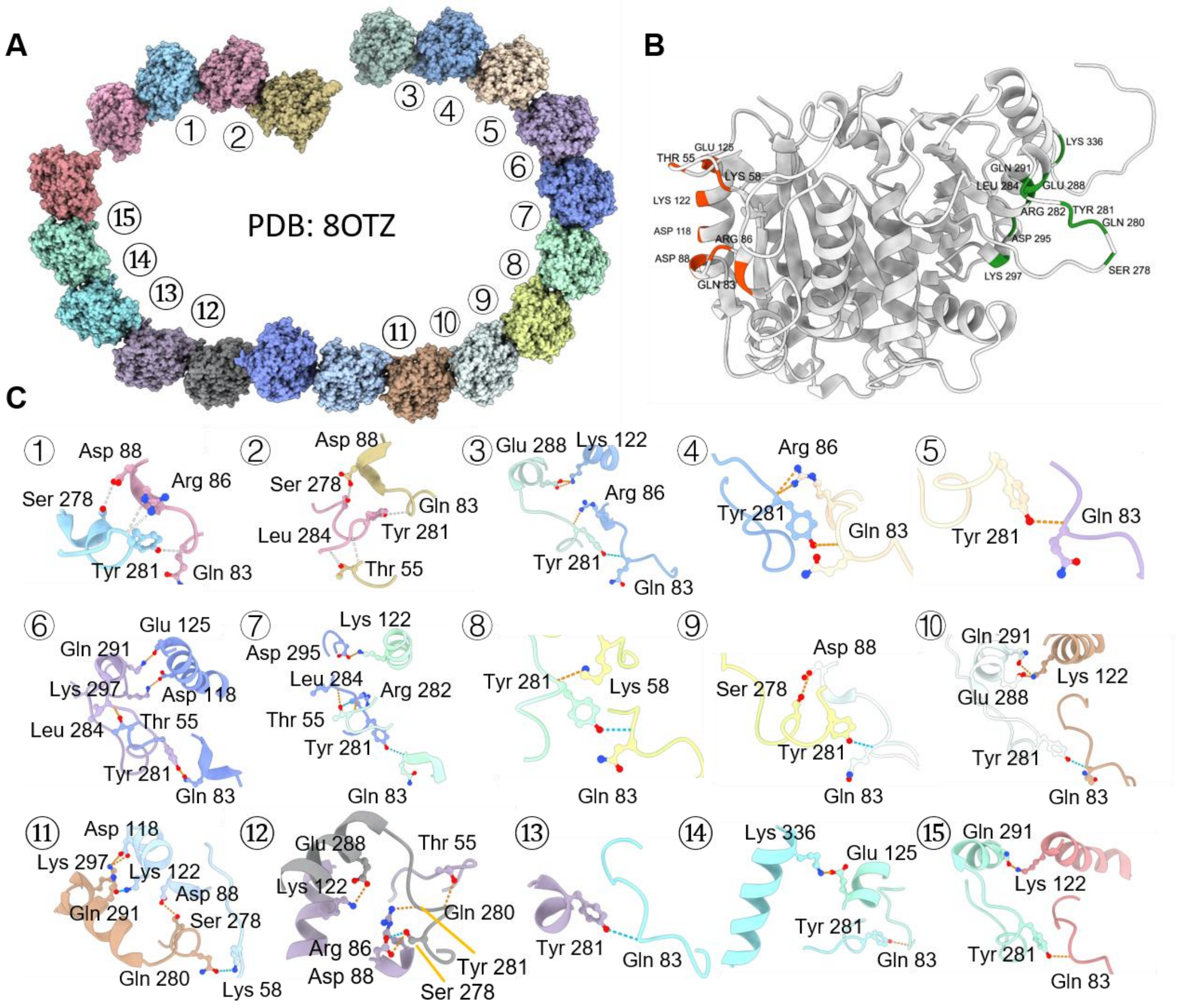

Figure S2. Lateral hydrogen-bonding interfaces in β-tubulin. *A*, A single layer of β-tubulin subunits extracted from the cryo-EM structure (PDB ID: 8OTZ), with the interfaces between adjacent β-tubulin subunits labeled 1–15. *B*, Structural mapping of residues involved in lateral hydrogen bonding with neighboring tubulin subunits, corresponding to the interactions shown in panel (*C*). *C*, Fifteen sets of lateral hydrogen-bonding interactions identified at the interfaces, with numerical labels corresponding to those in panel (*A*).

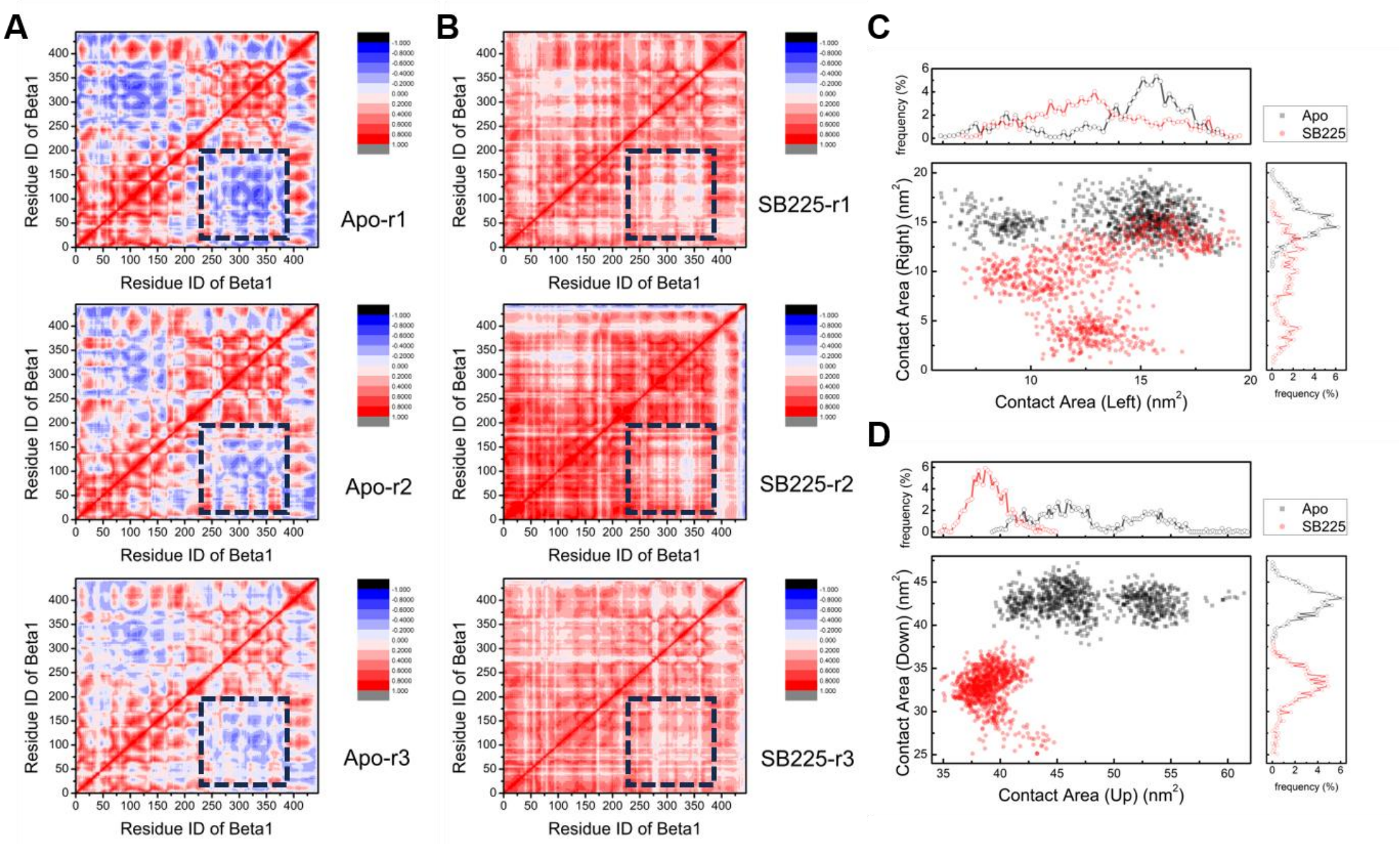

Figure S3. Independent dynamic cross-correlation matrices (DCCM) analyses and uncombined contact-area distributions. (*A*, *B*) DCCMs of β-tubulin obtained from three independent simulations under apo (*A*) and drug-bound (*B*) conditions. *C*, Frequency distributions of the uncombined contact areas between the middle column and the left and right adjacent columns in the 3 × 3 tubulin model. *D*, Frequency distributions of the uncombined contact areas between the central β-tubulin row and the upper and lower adjacent rows.

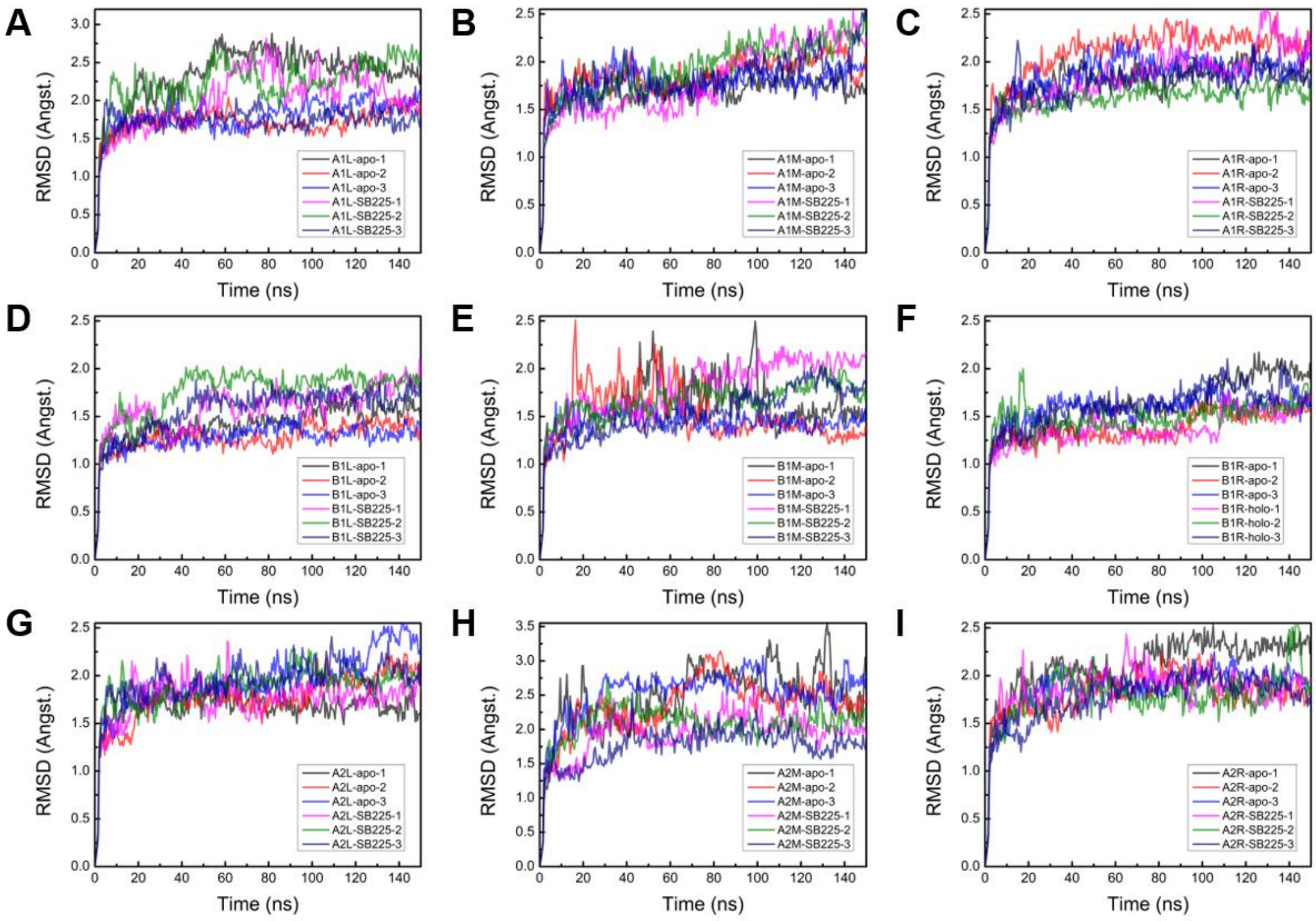

Figure S4. Cα root-mean-square deviation (RMSD) profiles of individual tubulin subunits in the 3 × 3 models. Cα RMSD values of each tubulin subunit during three independent 150-ns simulations of the drug-free and drug-bound systems.

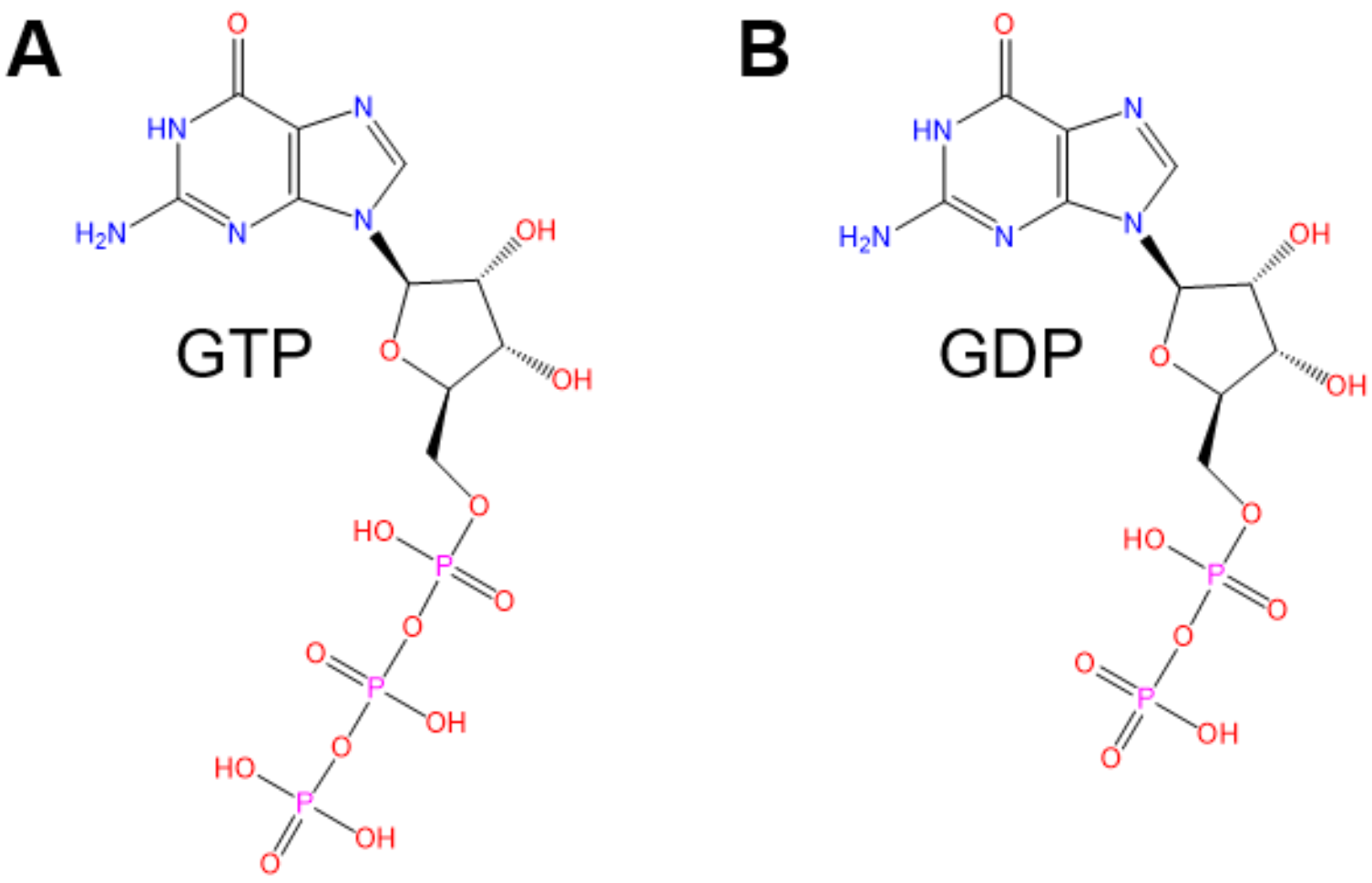

Figure S5. Chemical structures of tubulin-bound nucleotides. (*A*) Guanosine 5′-triphosphate (GTP). (*B*) Guanosine 5′-diphosphate (GDP).

MM/PBSA Calculation Parameters and Details

MM/PBSA calculations were performed using the CaFE package in VMD, with APBS employed for the Poisson–Boltzmann calculations (50). The key parameters used in the calculations are summarized in Table S1. A PB grid scale of 1.0 was used to balance computational efficiency and numerical accuracy. An effective internal dielectric constant of 30 was used for both the MM and PB calculations. To assess the sensitivity of the calculated energies to this parameter, preliminary calculations were performed using internal dielectric constants ranging from 1.5 to 40. Both the calculated energies and their associated uncertainties showed only minor changes at dielectric constants above 20 (Figure S6). Accordingly, a value of 30 was selected for the production calculations.

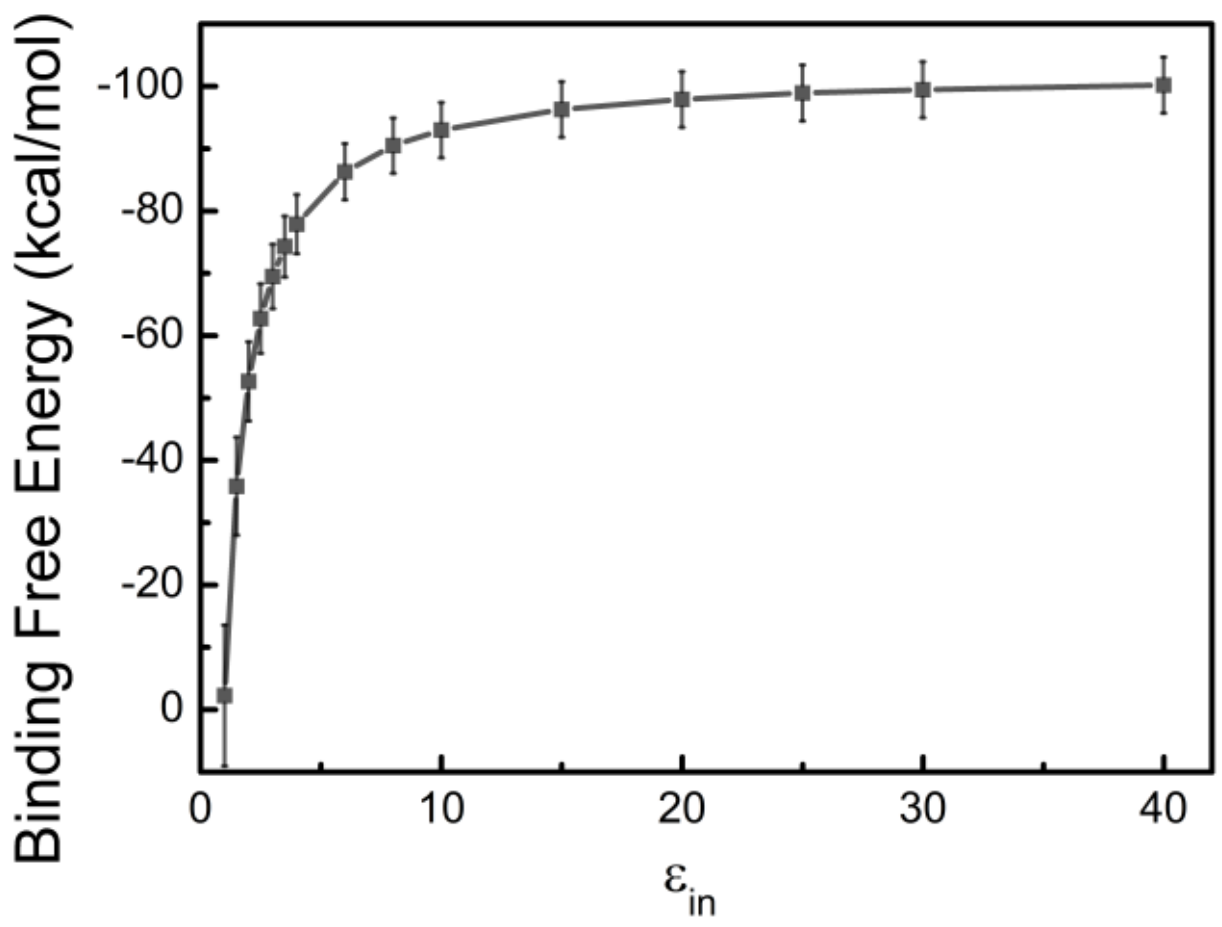

Figure S6. Effect of the internal dielectric constant on MM/PBSA-estimated binding free energies.

Table S1. Key parameters used in the MM/PBSA calculations.

Parameter	Value
PB solver	APBS
MM dielectric constant	30
PB internal dielectric constant	30
PB external dielectric constant	80
PB grid scale	1.0
PB radii	mparse
PB boundary condition	mdh
Charge mapping method	spl2
Surface tension coefficient	0.00542
Surface offset	0.92
Probe radius	1.4 Å

50. E. Jurrus, D. Engel, K. Star, K. Monson, J. Brandi, L.E. Felberg, et al., Improvements to the APBS biomolecular solvation software suite, Protein Sci., **27**, 2018, 112–128.
